# Comparison of linear and nonlinear models in estimation of variance components for reproductive traits in Markhoz goats

**DOI:** 10.1016/j.vas.2026.100757

**Published:** 2026-07-02

**Authors:** Somayeh Teymouri, Amir Rashidi, Peyman Mahmoudi, Mohammad Razmkabir, Rostam Abdollahi Arpanahi

**Affiliations:** a Department of Animal Science, Faculty of Agriculture, University of Kurdistan, Sanandaj, Iran; b Department of Animal Science, College of Agriculture and Natural Resources, University of Tehran, Karaj, Iran

**Keywords:** Markhoz goats, Nonlinear models, Reproduction traits, Variance components

## Abstract

The objective of this study was to estimate (co)variance components and genetic parameters for litter size at birth (LSB), litter size at weaning (LSW), and kid mortality from birth to weaning in Markhoz goats. Data were obtained from the Markhoz Goat Breeding Station in Sanandaj, Iran, and comprised 3439 records for LSB and LSW and 4087 records for kid mortality, collected over a 21-year period. Birth year and dam age had significant effects on LSB and LSW (*P* < 0.05), while birth year, dam age, birth type, and sex significantly affected kid mortality (*P* < 0.05). Genetic analyses for LSB and LSW were performed using linear and Poisson models, whereas linear and probit models were applied to mortality. Model performance was evaluated using predictive ability and goodness-of-fit statistics based on mean squared error of prediction and correlations between observed and fitted values. Heritability estimates ranged from 0.02 to 0.04 for LSB, 0.02 to 0.05 for LSW, and 0.16 to 0.35 for mortality. Estimated random effects, breeding values, and permanent environmental effects were highly correlated across models. For LSB, the linear model outperformed the Poisson model; for LSW, both models showed a similar fit, although the linear model had better predictive ability. For mortality, the linear model showed superior predictive performance, although the probit model uniquely identified maternal genetic and common litter effects. Overall, linear models are recommended for accurate animal ranking, whereas the probit model is preferred when the objective is to partition variance in binary traits into direct genetic, maternal, and litter components.

## 1 Introduction

Goats play a significant role in the agriculture of developing countries, where 90% of the world's goat population is raised (Hegde, 2020). Goat farming is a multipurpose economic activity for the production of meat, milk, and fiber, although meat production remains the primary objective (Andre Mataveia et al., 2023). Goat meat is considered higher in quality due to its lower fat content and reduced levels of saturated fatty acids compared to some other species; consequently, the demand for goat meat is increasing (Mazhangara et al., 2019). The Markhoz goat is a multipurpose breed raised for fiber (mohair), milk, and meat production. Although its primary product is mohair, the underdevelopment of the local textile industry and the subsequent substitution with synthetic fibers have led breeders to focus more on meat and milk production (Rashidi et al., 2014).

Reproductive efficiency is one of the most important factors affecting output in domestic animals, and reproductive traits are key determinants of profitability in small ruminant farming (Assan, 2020). In animal breeding, many economically important traits, such as fertility, mortality, twinning rate, viability, and conformation, are recorded as discrete categories rather than continuous measurements. Although their observed expression is discrete, it is assumed that an underlying, unobservable liability for these traits follows a normal distribution and is influenced by genetic and environmental factors, similar to other quantitative traits (Gregory et al., 1997).

Accurate estimation of variance and covariance components for economically important traits, including reproductive traits, is essential for predicting breeding values, estimating genetic parameters, and monitoring genetic progress. These components are commonly estimated using methods based on the threshold model concept (Bennewitz et al., 2007), most of which rely on maximum‑likelihood approaches within linear mixed models, particularly REML (Hofer, 1998). Henderson’s mixed model is widely used, providing BLUEs for fixed effects and BLUPs for random effects; however, linear models do not account for the discrete nature of threshold traits. Threshold models instead assume an unobserved, normally distributed variable that is categorized into discrete classes (Meijering & Gianola, 1985). Although linear models are simpler to implement, the adoption of nonlinear models such as threshold models has historically been constrained by their computational demands. Advances in computing and software now allow wider use of threshold models for variance component estimation (Misztal, 2008; Ødegård et al., 2010).

Given that key economic traits, such as fertility, litter size, and viability are inherently discrete, their effective incorporation into selection indices depends on accurate estimates of genetic parameters and reliable prediction of breeding values. Genetic improvement programs also require precise estimation of variances and covariances among traits to optimize selection decisions (Abdollahi-Arpanahi et al., 2013).

Several previous studies have compared linear and nonlinear models for discrete reproductive traits in livestock. Meijering and Gianola (1985) conducted simulation studies demonstrating that linear and nonlinear sire evaluation methods can yield different rankings for categorical traits. Peñagaricano et al. (2011) compared Poisson, probit, and linear models for genetic analysis of the presence and number of black spots in Corriedale sheep, finding that model choice substantially affected variance component estimates. Abdollahi-Arpanahi et al. (2013) similarly compared these three modeling frameworks for fertility traits in Iranian Holstein cows and reported that linear models provided competitive predictive performance despite the discrete nature of the traits. Ventura et al. (2015) compared Poisson and Gaussian Bayesian models for litter traits in pigs and highlighted the importance of accounting for overdispersion in count data. Bennewitz et al. (2007) applied Bayesian threshold models to reproductive traits in laying hens and demonstrated the utility of nonlinear approaches for partitioning genetic variance in binary traits. Despite these contributions, no study to date has systematically evaluated and compared linear, Poisson, and probit models for the estimation of variance components of litter size and early-life mortality traits specifically in Markhoz goats, an endangered, locally adapted breed with unique productive and demographic characteristics. Furthermore, prior studies have rarely assessed model performance using both goodness-of-fit statistics and cross-validation-based predictive ability simultaneously, limiting the ability to make practical recommendations for breeding program design. Therefore, this study addresses a critical gap by evaluating and comparing linear and nonlinear (Poisson and probit) models for estimating variance components of litter size at birth, litter size at weaning, and kid mortality from birth to weaning in Markhoz goats. We hypothesized that nonlinear models would yield higher heritability estimates and provide a more detailed partitioning of genetic and environmental variance for discrete traits, whereas linear models would demonstrate superior predictive performance and stability in cross-validation analyses. The results are expected to provide essential breed-specific insights for improving genetic evaluation strategies and enhancing conservation and selection programs for this economically important breed.

## 2 Material and methods

### 2.1 Data and traits

In this study, linear, Poisson, and probit models were applied to estimate the variance components of key reproductive traits in Markhoz goats. Model comparisons for litter size at birth (LSB), litter size at weaning (LSW), and kid mortality from birth to weaning were conducted using 3439 records for LSB and LSW and 4087 records for mortality. These data were collected over a 21-year period at the Sanandaj Markhoz Goat Breeding Station. The dataset included complete pedigree information, birth year, sex, birth type, dam age at kidding, and individual trait records.

### 2.2 Data processing

Microsoft Excel was used to organize and prepare the data file for analysis. For each animal, the dataset contained kid ID, sire ID, dam ID, birth year, sex, birth type, dam age at kidding, and the corresponding trait records. The GLM procedure in SAS (version 8.02) was used to identify significant fixed effects for inclusion in the statistical models. The datasets were then formatted for genetic analysis using the MCMCglmm package in the R programming environment.

### 2.3 Statistical models

The linear predictors for the traits analyzed were defined as follows:

$$\text{LSB}\;\text{and}\;\text{LSW}:\eta =Xb+Z_{a}a+Z_{pe}pe+e$$

$$\text{Mortality}:\;\eta =Xb+Z_{a}a+Z_{m}m+Z_{1}l+e$$

where η represents the linear predictor corresponding to the expected value of each trait (i.e., mortality or the number of kids born or weaned per doe); b represents the vector of fixed effects for year (21 levels), sex (2 levels), birth type (3 levels), and dam age (6 levels); a represents the vector of random additive genetic (animal) effects; m represents the vector of random maternal additive genetic effects (for mortality); pe is the vector of random maternal permanent environmental effects (for LSB and LSW); l is the vector of random common litter/environmental effects (for mortality); and e is the vector of random residual effects. X, Zₐ, Zₚₑ, Zₘ, and Zₗ are design matrices that relate the fixed and random effects to the observation vector. All random effects were assumed to follow multivariate normal distributions:

For LSB and LSW traits:

$$\left(\begin{array}{c} a\\ pe \end{array}\right)∼N\left[0\left(\begin{array}{cc} A\sigma_{a}^{2}\; & 0\\ 0\; & I\sigma_{\text{pe}}^{2}\; \end{array}\right)\right]$$

For the Mortality trait:

$$\left(\begin{array}{c} a\\ m\\ l \end{array}\right)∼N\left[0\left(\begin{array}{c} A\sigma_{a}^{2}\;0\;0\;\\ 0\;A\sigma_{m}^{2}\;0\;\\ 0\;0\;I\sigma_{l}^{2} \end{array}\right)\right]$$

where a, pe, m, and l represent the vectors of direct additive genetic effects, maternal permanent environmental effects, maternal additive genetic effects, and common litter effects, respectively; while $\sigma_{a}^{2}$, $\sigma_{\text{pe}}^{2}$, $\sigma_{m}^{2}$, and $\sigma_{l}^{2}$ denote the variances of direct additive genetic, maternal permanent environment, maternal additive genetic, and litter effects, respectively. Furthermore, A is the numerator relationship matrix, and I is an identity matrix. The additive genetic, maternal permanent environmental, maternal additive genetic, and common litter effects were assumed to be independent of the residual effects.

For simplicity and to avoid overparameterization given the available dataset size, the covariance between direct additive genetic effects (a) and maternal additive genetic effects (m) was assumed to be zero ($\sigma_{am}=0$). This assumption should be revisited in future studies with larger datasets that provide sufficient power to estimate this parameter reliably.

#### 2.3.1 Probit model

The probit model characterizes the relationship between a binary response variable and a set of quantitative or qualitative predictor variables. It is specifically suited for situations in which the outcome of interest is dichotomous (e.g., presence vs. absence of a trait or healthy vs. diseased status). In such analyses, the response is typically coded as 1 or 0, indicating the presence or absence of the phenotype, respectively. This modeling framework is applicable to any trait expressed in two distinct states, such as pregnancy status, occurrence of a physical mark, disease incidence, or survival versus mortality at birth. The essential requirement for applying a probit model is that the trait must be inherently binary.

In this study, kid mortality until weaning was treated as a binary variable, denoted as Z, where Z = 1 indicates death and Z = 0 indicates survival.

The probit model (Gianola, 1982) describes the observable outcomes using an underlying linear model:

z=η+e

where e is a vector of independent and identically distributed standard normal random variables. Therefore, the conditional probability of mortality is:

p(Mortality=1|η)=Φ(η)

where Φ(η) is the cumulative distribution function of the standard normal distribution. Accordingly, the likelihood function for the binary mortality data is expressed as:

$$p\left(\text{Mortality}|\eta \right)=\prod \Phi {\left(\eta \right)}^{\text{Mortality}}{\left[1-\Phi \left(\eta \right)\right]}^{1-\text{Mortality}}$$

where Mortality and η are vectors of binary outcomes and linear predictors, respectively, and Π the product over all observations.

#### 2.3.2 Poisson model

The Poisson distribution is commonly used to model the relative frequency of rare count events. It is applied to discrete count data to estimate the probability of events occurring within a defined time period, volume, or area. Examples include the number of lambs born per ewe or the number of colored spots on an animal’s body, both of which may follow a Poisson distribution (Kaps & Lamberson, 2004).

The Poisson model is represented by a single parameter λ, which denotes both the expected value and the variance of the random variable. Introducing a residual term allows modeling of individual differences in fit and overdispersion. A mixed Poisson model was assumed for LSB and LSW traits, incorporating the fixed and random effects described above. Under this framework, the Poisson distribution with parameter λ is defined such that:

log(λ)=η+e

where η is the linear predictor and e is a residual term assumed to follow an independent and identically distributed normal distribution, N (0,$\;\sigma_{e}^{2}$). Based on this formulation, the likelihood function for the count data is:

$$p\left\{\text{LSB}=t|\lambda \right\}=\prod \frac{\lambda^{t}\;\text{exp}\left\{-\lambda \right\}}{\;t!}$$

$$p\left\{\text{LSW}=t|\lambda \right\}=\prod \frac{\lambda^{t}\;\text{exp}\left\{-\lambda \right\}}{\;t!}$$

where t is the observed count and λ is the Poisson parameter.

#### 2.3.3 Linear model

Linear mixed models were fitted for all three traits: LSB, LSW, and Mortality. In these models, a normally distributed residual term is added to the linear predictor, yielding:

y=η+ewhere y is the vector of observations for LSB, LSW, and Mortality, and e is a random variable assumed to follow a normal distribution with mean zero and variance $\sigma_{e}^{2}$. Under these assumptions, the likelihood function for each response variable is given by:

$$p\left\{\text{LSB}│\eta \right\}=\prod N\;\left(LSB│\eta \;\sigma_{e}^{2}\right)$$

$$p\left\{\text{LSW}│\eta \right\}=\prod N\;\left(LSW│\eta \;\sigma_{e}^{2}\right)$$

$$p\left\{\text{Mortality}│\eta \right\}=\prod N\;\left(\text{Mortality}│\eta \;\sigma_{e}^{2}\right)$$

In the above equations, η represents the phenotype predicted using the linear models.

### 2.4 Statistical inference

According to Bayes' rule, the posterior density of the unknown model parameters is:

p(θ|y)∝p(y|θ)p(θ)where θ = {β₀, …, a, pe, m, l, $\sigma_{a}^{2}$, $\sigma_{\text{pe}}^{2}$, $\sigma_{m}^{2}$, $\sigma_{l}^{2}$, $\sigma_{e}^{2}$,} is the set of unknown model parameters, p(θ) is the joint prior distribution, p(y|θ) is the conditional distribution of the data (the likelihood function when viewed as a function of θ), and p(θ|y) is the posterior distribution of the unknowns. The joint prior distribution for the unknowns is specified as follows:

For LSB and LSW traits (Abdollahi et al., 2013; Penagaricano et al., 2011):

$$p\left(\theta \right)\propto p\left(a|\sigma_{a}^{2}\right)p\left(pe|\sigma_{pe}^{2}\right)p\left(\sigma_{a}^{2}\right)p\left(\sigma_{pe}^{2}\right)p\left(\sigma_{e}^{2}\right)$$

$$N\left(a|0,A\sigma_{a}^{2}\right)N\left(pe|0,A\sigma_{pe}^{2}\right)\chi^{-2}$$

$$\left(\sigma_{a}^{2}|df_{a},S_{a}\right)\chi^{-2}\left(\sigma_{pe}^{2}|df_{pe},S_{pe}\right)\chi^{-2}$$

$$\left(\sigma_{e}^{2}|df_{e},S_{e}\right)$$

For mortality:

$$p\left(\theta \right)\propto p\left(a|\sigma_{a}^{2}\right)p\left(m|\sigma_{m}^{2}\right)p\left(l|\sigma_{l}^{2}\right)p\left(\sigma_{a}^{2}\right)p\left(\sigma_{m}^{2}\right)p\left(\sigma_{l}^{2}\right)p\left(\sigma_{e}^{2}\right)$$

$$N\left(a|0,A\sigma_{a}^{2}\right)N\left(m|0,A\sigma_{m}^{2}\right)N\left(l|0,A\sigma_{l}^{2}\right)\chi^{-2}$$

$$\left(\sigma_{a}^{2}|df_{a},S_{a}\right)\chi^{-2}\left(\sigma_{m}^{2}|df_{m},S_{m}\right)\chi^{-2}\left(\sigma_{l}^{2}|df_{l},S_{l}\right)\chi^{-2}$$

$$\left(\sigma_{e}^{2}|df_{e},S_{e}\right)$$

where χ²(σ²|df., S.) denotes a scaled inverse chi-squared distribution with degrees of freedom df and scale parameter S.

### 2.5 Model implementation

For the linear, Poisson, and probit models, posterior distribution sampling was performed using the MCMCglmm package (Hadfield, 2010) in the R environment (R Core Development Team, 2021). Results for each trait were obtained based on 200,000 samples after discarding the initial 50,000 samples as burn-in. A sampling interval (thinning) of 50 was used for posterior distribution computation. Convergence diagnosis and statistical and graphical analysis of the Markov Chain Monte Carlo (MCMC) output were performed using the coda package (Plummer et al., 2006) in R. The Raftery and Lewis (1992) method and visual inspection of posterior distribution plots were used to assess the convergence of the MCMCglmm outputs. Effective sample sizes (ESS) for all variance components were acceptable, with most values exceeding 400 (range: 388–1832). All Raftery‑Lewis dependence factors were below 1.2, indicating low autocorrelation. Together with visual inspection of trace plots, these diagnostics confirm that the chosen burn‑in (50,000) and thinning interval (50) were sufficient to obtain reliable posterior summaries. Detailed diagnostics, including ESS and trace plots, are provided in Supplementary Table S1 and Supplementary File S1.

### 2.6 Estimation of genetic parameters

In the linear and Poisson models, heritability (h²) and repeatability (r) can be estimated on the observable and logarithmic scales, respectively, using standard formulas:

For LSB and LSW traits:

$$\text{Heritability}:h^{2}=\frac{\sigma_{a}^{2}}{\sigma_{a}^{2}+\;\sigma_{pe}^{2}+\;\sigma_{e}^{2}}$$

$$\text{Repeatability}:r=\frac{\sigma_{a}^{2}+\;\sigma_{pe}^{2}}{\sigma_{a}^{2}+\;\sigma_{pe}^{2}+\;\sigma_{e}^{2}}$$

For the Mortality trait:

$$\text{Heritability}:\;h^{2}=\frac{\sigma_{a}^{2}}{\sigma_{a}^{2}+\;\sigma_{m}^{2}+\sigma_{l}^{2}+\;\sigma_{e}^{2}}$$

In the probit model, h² and r can be evaluated on the underlying liability scale using the formulas above, considering $\sigma_{e}^{2}=1$ (Abdollahi-Arpanahi et al., 2013; Peñagaricano et al., 2011).

### 2.7 Model comparison

The goodness of fit for each model was evaluated by estimating the Mean Squared Error (MSE):

$$MSE=n^{-1}\sum {\left(y-\hat{y}\right)}^{2}$$

where ŷ is the conditional expectation function (predicted value) evaluated at the posterior mean of the model unknowns, y is the corresponding vector of observed values (for LSB, LSW, and Mortality), and n is the number of observations. The conditional expectation functions for models were:

$$\text{Linear}:\hat{y}=\hat{\eta }$$

$$\text{Poisson}:\;\hat{y}=\theta \left(\hat{\eta }\right)$$

Probit: $\hat{y}=\Phi \left(\hat{\eta }\right)$, where Φ is the cumulative distribution function of the standard normal distribution.

Models were also compared using the Spearman rank correlation between the predicted random effects obtained from different models. Random effects were predicted using their posterior mean estimates.

To complement the in-sample goodness-of-fit statistics, three-fold cross-validation with 10 replications was used to evaluate the out-of-sample predictive ability of each model. While in-sample metrics such as MSE reflect how well a model fits the data used for its own estimation, cross-validation assesses how well a model predicts new, unseen observations, a more relevant criterion for practical genetic evaluation, where breeding values estimated from historical records must predict future offspring performance. This distinction is especially important when comparing models of differing complexity and distributional assumptions. In this cross-validation method, the dataset was divided into three equal parts while ensuring that data were uniformly distributed across the levels of fixed effects in both the training and testing sets. Solutions for all fixed and random effects were then estimated using the training set (two folds) and used to predict observations in the testing set (the remaining fold). The MSE and correlation between observed and fitted values were calculated. The data partitioning was also repeated based on the number of records per animal to evaluate the predictive ability of each model under different levels of data availability.

The prediction mean squared error (MSEP) was calculated following Abdollahi-Arpanahi et al. (2013) and Peñagaricano et al. (2011) and defined as:

$$MSEP=n^{-1}\;\sum_{f}\sum {\left(y-\;{ŷ}^{f}\right)}^{2}$$

where ŷᶠ denotes the conditional expectation (predicted phenotypic value) evaluated at the posterior mean of the model parameters when the observations in fold f were excluded from the analysis. This cross-validation framework allows assessment of each model’s predictive ability. In addition, the Pearson correlation between observed and fitted values, along with the MSEP in the testing set, was estimated to further compare predictive performance across models.

## 3 Results

### 3.1 Descriptive statistics of reproductive and mortality traits

Descriptive statistics for the reproductive and mortality traits are presented in Table 1. The number of kids born per doe per kidding (LSB) had 3439 records, with values ranging from 1 to 3, a mean of 1.28, and a standard deviation of 0.46, corresponding to a coefficient of variation of 36%. The number of kids weaned per doe per kidding (LSW), also based on 3439 observations, ranged from 0 to 3, with a mean of 1.07 and a standard deviation of 0.62, yielding a coefficient of variation of 58%. Kid mortality from birth to weaning was recorded for 4087 observations, ranged from 0 to 1, had a mean of 0.12 and a standard deviation of 0.31, and resulted in a coefficient of variation of 258%.

**Table 1:** Descriptive statistics of data related to reproductive and mortality traits.

Traits^a^	Parameters^b^					
	N	Min	Max	Mean	SD	CV (%)
LSB	3439	1	3	1.28	0.46	36
LSW	3439	0	3	1.07	0.62	58
Mortality	4087	0	1	0.12	0.31	258

a LSB: Number of kids born per doe kidding, LSW: Number of kids weaned per doe kidding, Mortality: Kid mortality from birth to weaning.

b N: Number of observations, Min: Minimum, Max: Maximum, SD: Standard deviation, CV: Coefficient of variance.

### 3.2 Pedigree structure

The data and pedigree structure for the reproductive and mortality traits are summarized in Table 2. For LSB and LSW, the pedigree included 1548 animals, consisting of 180 sires and 660 dams, with an average of 8.60 progeny per sire. A total of 1063 animals had both parents identified, and no animals had only one known parent. For the mortality trait, the pedigree comprised 4558 animals, including 198 sires and 1325 dams, with an average of 23.02 progeny per sire. Of these animals, 4066 had both parents known, while 21 had one unknown parent.

**Table 2:** Data and pedigree structure for reproductive and mortality traits.

Item	Traits^a^	
	LSB and LSW	Mortality
Number of animals in pedigree	1548	4558
Number of sires in pedigree	180	198
Number of dams in pedigree	660	1325
Number of progenies per sire	8.60	23.02
Number of animals with both parents known	1063	4066
Number of animals with one unknown parent	0	21

a For trait abbreviations see footnote of Table 1.

### 3.3 Frequency distribution of kid birth types

The frequency distribution of birth types is presented in Table 3. Among all recorded births, single births were the most common, accounting for 2473 cases (71.95%). Twin births represented 942 cases (27.39%), while triplet births were rare, occurring in only 24 cases (0.69%). The overall multiple birth rate was 16.2%, with twins comprising the majority of multiple births and triplets contributing only 0.2%.

**Table 3:** Frequency distribution of birth types for kids.

Birth Type	Frequency	Birth Type (%)	Multiple Birth Rate (%)
Single	2473	71.95	83.8
Twin	942	27.39	16.0
Triplet	24	0.69	0.2

### 3.4 Estimation of fixed effects on reproductive traits

The fixed effects analysis revealed that year of kidding had a significant effect (*P* < 0.01) on all three traits: litter size at birth (LSB), litter size at weaning (LSW), and kid mortality. Age of the dam also significantly influenced all three traits (*P* < 0.01). Sex of the kid did not significantly affect LSB or LSW but showed a significant effect on mortality (*P* < 0.01), with male kids exhibiting higher mortality rates. Similarly, type of birth (single, twin, triplet) did not significantly affect LSB or LSW but had a significant effect on mortality (*P* < 0.01), where multiple-born kids faced greater survival challenges.

### 3.5 Estimation of variance components and genetic parameters

The estimates of variance components and genetic parameters for LSB, LSW, and kid mortality under different statistical models are summarized in Table 4. For LSB and LSW, the Poisson model yielded higher additive genetic variances (0.013 and 0.01, respectively) compared with the linear model (0.004 for both traits). Permanent environmental variance was also greater under the Poisson model (0.03) than under the linear model (0.01). Consequently, phenotypic variances were higher in the Poisson model (0.25 for both traits) than in the linear model (0.19 for LSB and 0.18 for LSW). Heritability estimates for these traits were low across models, ranging from 0.02 to 0.04 for LSB and 0.02 to 0.05 for LSW. Repeatability estimates were similarly low, varying from 0.07 to 0.16 for LSB and 0.08 to 0.17 for LSW. For kid mortality, the probit model produced markedly higher additive genetic (0.84) and phenotypic (2.38) variances compared with the linear model (0.016 and 0.10, respectively). Maternal genetic and litter environmental variances were detected only in the probit model. Heritability estimates for mortality were moderate: 0.16 under the linear model and 0.35 under the probit model.

**Table 4:** Estimation of variance component and genetic parameters based on different models.

Variance Component b	Traits a					
	LSB		LSW		Mortality	
	Linear	Poisson	Linear	Poisson	Linear	Probit
$\sigma_{a}^{2}$	0.004 ± 0.001	0.013 ± 0.005	0.004 ± 0.001	0.01 ± 0.004	0.016 ± 0.003	0.84 ± 0.14
$\sigma_{pe}^{2}$	0.01 ± 0.007	0.03 ± 0.01	0.01 ± 0.006	0.03 ± 0.008	-	-
$\sigma_{m}^{2}$	-	-	-	-	0.003 ± 0.001	0.17 ± 0.04
$\sigma_{l}^{2}$	-	-	-	-	0.01 ± 0.007	0.37 ± 0.12
$\sigma_{e}^{2}$	0.18 ± 0.03	0.21 ± 0.04	0.17 ± 0.05	0.21 ± 0.05	0.07 ± 0.02	1.00 ± 0.23
$\sigma_{P}^{2}$	0.19 ± 0.04	0.25 ± 0.04	0.18 ± 0.06	0.25 ± 0.04	0.10 ± 0.03	2.38 ± 0.76
h^2^	0.02 ± 0.02	0.04 ± 0.01	0.02 ± 0.02	0.05 ± 0.02	0.16 ± 0.03	0.35 ± 0.05
r	0.07 ± 0.02	0.16 ± 0.03	0.08 ± 0.03	0.17 ± 0.03	-	-

a : For trait abbreviations see footnote of Table 1.

b :$\;\sigma_{a}^{2}:$ Additive genetic variance $\sigma_{pe}^{2}$: Permanent environmental variance $\sigma_{m}^{2}:\;\text{Maternal}\;\text{genetic}\;\text{variance}$$\sigma_{l}^{2}$: Common environmental variance (litter effect) $\sigma_{e}^{2}$: Residual variance $\sigma_{P}^{2}$: Phenotypic variance, h²: Direct heritability, r: Repeatability.

### 3.6 Correlations among breeding values and permanent environmental effects

**Fig. 1:**
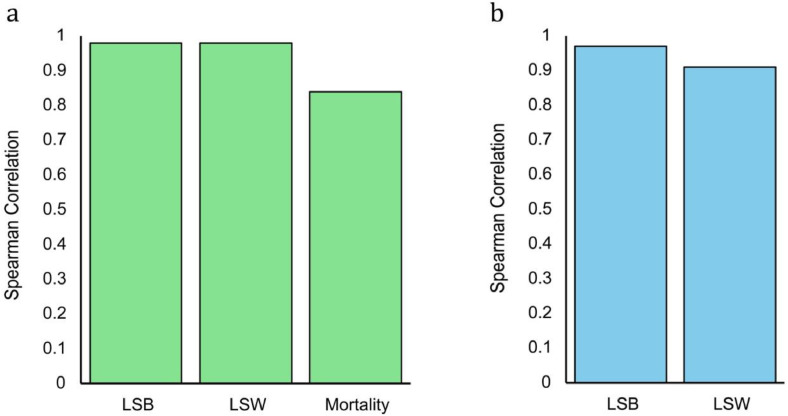
Spearman rank correlations between random effects estimated from different statistical models for reproductive traits in Markhoz goats. (a) Correlations between breeding values (BV) obtained from linear vs. Poisson models for litter size at birth (LSB) and litter size at weaning (LSW), and from linear vs. probit models for kid mortality. (b) Correlations between permanent environmental effects (Pe) obtained from linear vs. Poisson models for LSB and LSW. High correlations (≥0.91) indicate strong agreement in animal ranking across models, supporting the robustness of genetic evaluations irrespective of the underlying distributional assumptions.

Analysis of correlations among breeding values (BV) and permanent environmental effects (Pe) obtained from different statistical models is shown in Fig. 1a and b. Results showed consistently high agreement across models for all traits. For BV, the linear and Poisson models produced identical selections for litter size at birth (LSB) and litter size at weaning (LSW), both yielding very strong Spearman correlations of 0.98 (Fig. 1a). Mortality showed a slightly lower but still strong correlation of 0.84 between the linear and probit models. For Pe effects, correlations among models also remained high, with LSB showing a correlation of 0.97 between linear and Poisson models, while LSW showed a correlation of 0.91 (Fig. 1b). Overall, these results indicate substantial consistency between different modeling approaches in ranking animals for both genetic and permanent environmental components of reproductive traits.

### 3.7 Cross-validation assessment of model predictive ability

The predictive performance of the linear and Poisson models for LSB and LSW across different cross-validation groups is shown in Table 5. Results demonstrated moderate variation depending on the number of records per individual. When all data were included in the test set, the linear model showed slightly superior predictive accuracy for both traits, with lower MSEP values (0.178 for LSB and 0.276 for LSW) and higher COR values (0.458 and 0.559, respectively) compared with the Poisson model. Under the N = 1 category, where only one observation per animal was used, the linear model again exhibited lower error for LSB (MSEP = 0.141) than the Poisson model (0.166), while the Poisson model provided a marginally stronger correlation (0.446 vs. 0.350). A similar trend was observed for LSW, where the linear model resulted in lower MSEP (0.257 vs. 0.284) and higher COR (0.482 vs. 0.457) compared to the Poisson analysis. When the number of records increased to N = 2, linear modeling maintained lower prediction error (MSEP = 0.174 for LSB and 0.299 for LSW) and also yielded a higher correlation for LSB (0.364) compared with the Poisson model (0.259). For animals with more than two records (N > 2), linear models consistently outperformed the Poisson model, with notably lower MSEP values (0.181 vs. 0.200 for LSB; 0.273 vs. 0.283 for LSW) and stronger predictive correlations (0.474 vs. 0.361 for LSB; 0.578 vs. 0.531 for LSW). Overall, these results indicate that predictive accuracy improved as the number of observations per animal increased, and linear models generally provided more robust predictive performance for the reproductive traits evaluated.

**Table 5:** Mean squared error of prediction (MSEP) and Pearson correlation (COR) between observed and predicted values across three cross-validation groups for each trait and model.

Methods	Parameters	Traits a					
		LSB		LSW		Mortality	
		Linear	Poisson	Linear	Poisson	Linear	Probit
All data in the test group	MSEP	0.178	0.196	0.276	0.287	0.092	0.103
	COR	0.458	0.356	0.559	0.513	0.254	0.142
N = 1	MSEP	0.141	0.166	0.257	0.284	0.086	0.089
	COR	0.350	0.446	0.482	0.457	0.214	0.178
N = 2	MSEP	0.174	0.182	0.299	0.308	0.081	0.086
	COR	0.364	0.259	0.429	0.401	0.198	0.204
N > 2	MSEP	0.181	0.200	0.273	0.283	0.095	0.112
	COR	0.474	0.361	0.578	0.531	0.247	0.228

a : For trait abbreviations see footnote of Table 1.

## 4 Discussion

The results of this study indicate that several fixed effects play important roles in shaping reproductive performance and early-life survival in the Markhoz goat population. The significant effect of kidding year on litter size at birth (LSB), litter size at weaning (LSW), and mortality suggests that temporal and environmental fluctuations substantially influence reproductive outcomes. Yearly variations may reflect differences in management practices, feed availability, climate conditions, or disease pressures, all of which can affect both dam productivity and kid survival. These findings are consistent with Zhang et al. (2009), who reported a significant effect of birth year on LSB and LSW in Boer goats, and Kebede et al. (2012), who observed a similar effect in Arsi-Bale goats.

The age of the dam also had a significant effect on all traits examined, highlighting the biological and physiological differences across parity groups. Younger dams often have lower reproductive efficiency due to incomplete physiological maturity, whereas older, experienced dams typically demonstrate improved maternal abilities, larger litters, and better offspring survival (Vlahek et al., 2023). These results are consistent with findings in Raeini Cashmere goats (Mohammadi et al., 2012) and Saanen goats (Mawussi et al., 2024). Similarly, Zhang et al. (2009) reported a significant effect of age on these traits in Boer goats.

In contrast, the sex of the kid did not significantly influence litter size traits (LSB or LSW), which is expected because these traits are determined before birth and are not influenced by the sex of individual offspring. However, sex had a significant effect on mortality, suggesting that male and female kids may differ in vulnerability during early life. Male offspring may be more susceptible to environmental stress or disease due to physiological or behavioral differences (Sutherland & Brunwasser, 2018). These results are consistent with the findings of Al-Khaza’leh et al. (2020), who reported in a study on small ruminants in Jordan that the mean annual mortality rates of male kids and lambs were significantly higher than those of females. Furthermore, Chauhan et al. (2019) determined that male Sirohi goat kids had higher hazards of death compared with female kids, based on a multivariable Cox regression analysis of survival from birth to weaning.

Similarly, birth type did not affect LSB or LSW but significantly influenced mortality. Single-born kids generally experience less competition for maternal resources and have higher survival rates, whereas twins or multiples are more prone to lower birth weight and reduced vitality. The significant effect of birth type on mortality is therefore consistent with biological expectations and corroborates previous research indicating that multiple-born kids face greater survival challenges (Chauhan et al., 2019; Yitagesu & Alemnew, 2022).

As expected for count traits, the Poisson model produced higher additive genetic and permanent environmental variances than the linear model (Table 4), because linear models underestimate variance for discrete distributions (Meijering & Gianola, 1985).

Heritability estimates for litter size were low (0.02–0.05) and consistent with values reported for other small ruminants (Vázquez-Armijo et al., 2021; Ziadi et al., 2021), indicating that environmental and physiological factors dominate over additive genetic effects. Low repeatability further suggests that management improvements may yield faster gains than selection.

Furthermore, the assumption of zero direct-maternal genetic covariance ($\sigma_{am}=0$) in the mortality model, while necessary for model parsimony given the dataset size, represents a potential source of bias. A non-zero, potentially antagonistic, covariance between direct and maternal genetic effects has been reported for early survival traits in other ruminant species, and its omission may result in upward or downward bias in the individual variance component estimates. Future analyses with more extensive and multigenerational datasets should include this parameter.

The results for kid mortality illustrate the importance of using an appropriate statistical model for binary traits. The probit model produced markedly higher estimates of additive genetic and phenotypic variances compared with the linear model, highlighting the limitations of linear models for categorical survival traits (Carlin & Moreno‐Betancur, 2025). Importantly, maternal genetic and litter environmental effects were detected only in the probit model, demonstrating its superior capacity to partition sources of variation relevant to early-life survival. The higher heritability estimate obtained under the probit model (0.35) on the underlying liability scale suggests that genetic improvement in kid survival may be more feasible than indicated by the linear model (0.16). This moderate heritability implies that both direct genetic effects of the kid and maternal influences contribute meaningfully to survival outcomes.

High Spearman correlations between breeding values from different models (Fig. 1a) confirm that animal rankings are robust to model choice for both litter size (ρ = 0.98) and mortality (ρ = 0.84). Permanent environmental effects were also highly correlated across models (Fig. 1b), with the slightly lower value for LSW (ρ = 0.91) possibly reflecting greater postnatal environmental variation.

The correlation between the linear and probit models for kid mortality, although slightly lower (0.84), still reflects substantial agreement. Mortality is a binary trait that inherently benefits from threshold modeling, and some differences in predicted breeding values between linear and probit models are expected. Nonetheless, the strong positive correlation indicates that both methods similarly rank animals for survival potential, providing confidence in the reliability of selection decisions based on either model.

Permanent environmental effects also showed high correlations between the linear and Poisson models for LSB (0.97) and LSW (0.91). These results demonstrate that environmental influences attributable to individual dams are consistently estimated across models, reinforcing the stability of non-genetic evaluations. The slightly lower correlation for LSW likely reflects the greater sensitivity of weaning outcomes to postnatal management conditions, nutrition, and kid-specific factors (Wang et al., 2023).

The comparison of model performance across the evaluated traits reveals that linear modeling generally provides superior predictive capability relative to Poisson and probit alternatives. For the fecundity-related traits (LSB and LSW), the lower MSE values obtained from the linear model indicate a closer fit between observed and predicted outcomes, while the noticeably higher correlation coefficients reflect stronger consistency in the prediction pattern. These findings align with previous studies suggesting that continuous approximations may be more suitable for traits with moderate dispersion or when the variance does not strictly follow a Poisson distribution (Gianola & Fernando, 1986; Ventura et al., 2015). Although LSB and LSW represent count variables, their distributions in livestock populations are often over-dispersed, leading to reduced efficiency of classical Poisson models.

Similarly, for mortality, typically analyzed as a binary or categorical trait, the linear model again outperformed the probit model, though the differences were less pronounced. This suggests that even for threshold-type traits, linear assumptions may still capture a substantial portion of the phenotypic variability. The weaker performance of the probit model may also be attributable to limited variation in mortality records, which constrains the model’s ability to differentiate categorical outcomes effectively.

It should be noted that simultaneous estimation of maternal additive genetic (m) and common litter environmental (l) effects may be subject to partial confounding, particularly for dams with limited repeated records. Researchers should therefore interpret the partitioned variance components for these two effects with caution, and future studies with larger datasets and richer pedigree structures should revisit this decomposition.

The cross-validation outcomes for reproductive traits further support the superiority of linear modeling approaches in predictive performance, while also highlighting the influence of data availability per individual. Across all cross-validation structures, the linear model consistently yielded lower prediction error (MSEP) for both LSB and LSW. This suggests that the linear model is more effective at capturing the underlying variability of reproductive outcomes, even though these traits are count-based and inherently non-normal. These findings corroborate the argument that reproductive traits in livestock often show overdispersion and do not strictly follow a Poisson distribution, making linear approximations statistically more efficient in many practical breeding scenarios (Meijering & Gianola, 1985; Tempelman & Gianola, 1999).

Variability in prediction strength across animal record groups indicates that data completeness plays a crucial role in model reliability. When only a single observation per animal was available (N = 1), predictive ability was reduced for both models, as indicated by the lower correlation values. This diminished accuracy likely reflects the limited phenotypic information available to estimate individual deviation from the overall population mean. As the number of records increased (N = 2 and N > 2), both error measures and correlation coefficients improved substantially, confirming that repeated records enhance the separation of genetic and environmental components in animal models (Falconer, 1995).

Several limitations of this study should be acknowledged. First, the number of sires for the litter size traits (LSB and LSW) was relatively small (n = 180), which may reduce the precision of additive genetic variance estimates and limit the ability to detect small genetic effects. Second, the data originated from a single breeding station and a single breed (Markhoz goats). Therefore, the findings may not be directly generalizable to other goat populations, breeds, or management systems without further validation. Third, the potential influence of inbreeding was not accounted for in the models. Previous research on this same population (Rashidi et al., 2014) has shown that inbreeding coefficients can affect birth weight and kid survival, suggesting that unmodeled inbreeding could bias variance component estimates, particularly for mortality. Fourth, given the low heritabilities estimated for LSB and LSW (0.02–0.05), statistical power to discriminate between linear and Poisson models is inherently limited, larger datasets or repeated records per animal would be required to detect subtle differences in model fit with greater confidence. Additionally, the assumption of zero covariance between direct and maternal genetic effects ($\sigma_{am}=0$) was made to avoid overparameterization, but this may not hold biologically. Finally, the cross-validation design, while robust, was limited to the available data structure; independent validation on external datasets would strengthen confidence in the model comparisons.

Despite these limitations, the study provides the first systematic comparison of linear, Poisson, and probit models for reproductive traits in Markhoz goats, and the results offer practical guidance for genetic evaluation in this endangered breed.

## 5 Conclusions

This study provides the first systematic comparison of linear, Poisson, and probit models for genetic evaluation of reproductive traits in Markhoz goats. Based on our findings, linear models are recommended for predicting breeding values for litter size at birth, litter size at weaning, and kid mortality when the primary goal is accurate ranking of animals for selection. Linear models consistently gave lower cross-validation prediction errors (MSEP) and higher correlations between observed and predicted values, despite the discrete nature of the traits. Their computational simplicity and robustness to over-dispersion make them a practical choice for herd-level implementation. The probit model should be used when the objective is to partition variance for binary traits such as mortality into direct genetic, maternal genetic, and common litter components. The detection of significant maternal genetic variance and litter effects under the probit model, effects that were not estimable with the linear model, provides valuable insights for designing crossbreeding or management strategies to improve kid survival.

Genetic progress for LSB and LSW will be slow. Breeders should prioritize environmental and management improvements (nutrition, disease control, dam age structure) over intense selection for litter size. The low repeatability (0.07–0.17) further indicates that culling based on a single parity record is unreliable; repeated records are necessary to assess a doe’s genetic potential. The moderate heritability under the probit model (0.35) suggests that selective breeding could contribute to reduced mortality. However, maternal genetic effects and common litter effects also play substantial roles, implying that improving dam rearing ability and litter uniformity may be as important as direct genetic selection on the kid.

Future research directions should include: (i) estimation of the direct-maternal genetic covariance for mortality, which was assumed zero in this study due to dataset size; (ii) validation of these model comparisons in other goat populations and production systems; (iii) extension to multi-trait models that jointly analyze litter size and mortality to account for potential genetic correlations; (iv) longer MCMC chains or alternative priors to improve mixing for Poisson model variance components (e.g., residual ESS was low for some parameters); and (v) incorporation of inbreeding coefficients (known to affect survival in this breed) as a covariate in future genetic evaluations.

## Ethical statement

All data used in this study were obtained from existing records collected at the Sanandaj Markhoz goat Breeding Station. Since no new animals were used or subjected to experimental procedures, formal ethical approval was not required for this research.

## Funding

This research did not receive any specific grant from funding agencies in the public, commercial, or not-for-profit sectors.

## CRediT authorship contribution statement

**Somayeh Teymouri:** Writing – original draft, Investigation, Formal analysis. **Amir Rashidi:** Writing – review & editing, Supervision, Conceptualization. **Peyman Mahmoudi:** Writing – review & editing, Software, Formal analysis, Data curation. **Mohammad Razmkabir:** Writing – review & editing, Validation, Methodology. **Rostam Abdollahi Arpanahi:** Writing – review & editing, Validation, Methodology.

## Declaration of competing interest

The authors declare that they have no known competing financial interests or personal relationships that could have appeared to influence the work reported in this paper.

## Data Availability

The data that support the findings of this study are available from the corresponding authors upon reasonable request.

## Appendix Supplementary materials

Image, application 1
