# Supplementary material for: Comparison of linear and nonlinear models in estimation of variance components for reproductive traits in Markhoz goats
Source: Vet Anim Sci. 2026 Jul 2;34:100757. doi: 10.1016/j.vas.2026.100757 (PMC13382119; doi:10.1016/j.vas.2026.100757)
Supplement: Supplementary file 1 [file mmc1.docx]

| **Table S1.** Convergence diagnostics for MCMC samples of variance components | | | | |
| --- | --- | --- | --- | --- |
| Trait | Model | Parameter | ESS | Raftery-Lewis (dependence factor) |
| LSB | Linear | $\sigma_{a}^{2}$ | 598 | 1.12 |
|  |  | $\sigma_{pe}^{2}$ | 543 | 1.06 |
|  |  | $\sigma_{e}^{2}$ | 1832 | 1.04 |
|  | Poisson | $\sigma_{a}^{2}$ | 486 | 1.18 |
|  |  | $\sigma_{pe}^{2}$ | 407 | 1.08 |
|  |  | $\sigma_{e}^{2}$ | 538 | 1.05 |
| LSW | Linear | $\sigma_{a}^{2}$ | 541 | 1.08 |
|  |  | $\sigma_{pe}^{2}$ | 520 | 1.04 |
|  |  | $\sigma_{e}^{2}$ | 1418 | 1.01 |
|  | Poisson | $\sigma_{a}^{2}$ | 462 | 1.13 |
|  |  | $\sigma_{pe}^{2}$ | 388 | 1.07 |
|  |  | $\sigma_{e}^{2}$ | 614 | 1.06 |
| Mortality | Linear | $\sigma_{a}^{2}$ | 675 | 1.01 |
|  |  | $\sigma_{m}^{2}$ | 511 | 1.02 |
|  |  | $\sigma_{l}^{2}$ | 551 | 1.01 |
|  |  | $\sigma_{e}^{2}$ | 1126 | 1.01 |
|  | Probit | $\sigma_{a}^{2}$ | 573 | 1.12 |
|  |  | $\sigma_{m}^{2}$ | 504 | 1.15 |
|  |  | $\sigma_{l}^{2}$ | 485 | 1.14 |
|  |  | $\sigma_{e}^{2}$ | 966 | 1.09 |
| $\sigma_{a}^{2}:$ additive genetic variance; $\sigma_{pe}^{2}$: permanent environmental variance; $\sigma_{m}^{2}$: maternal genetic variance; $\sigma_{l}^{2}:$ common environmental (litter) variance; $\sigma_{e}^{2}:$ residual variance.  ESS: Effective Sample Sizes | | | | |


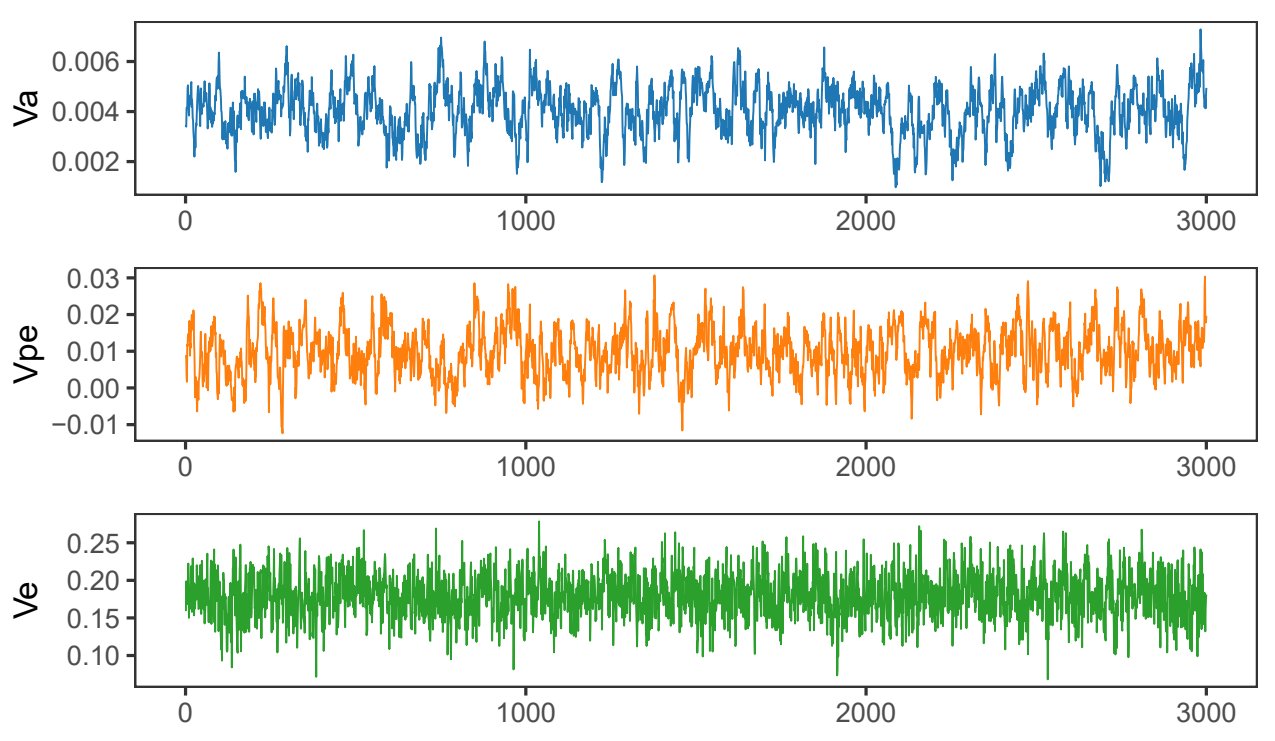


**Figure S1.** Trace plot of variance components for litter size at birth (LSB) from the linear model. $V_{a}$, blue: additive genetic variance; $V_{\mathrm{pe}}$, orange: permanent environmental variance; and $V_{e}$, green: residual variance.


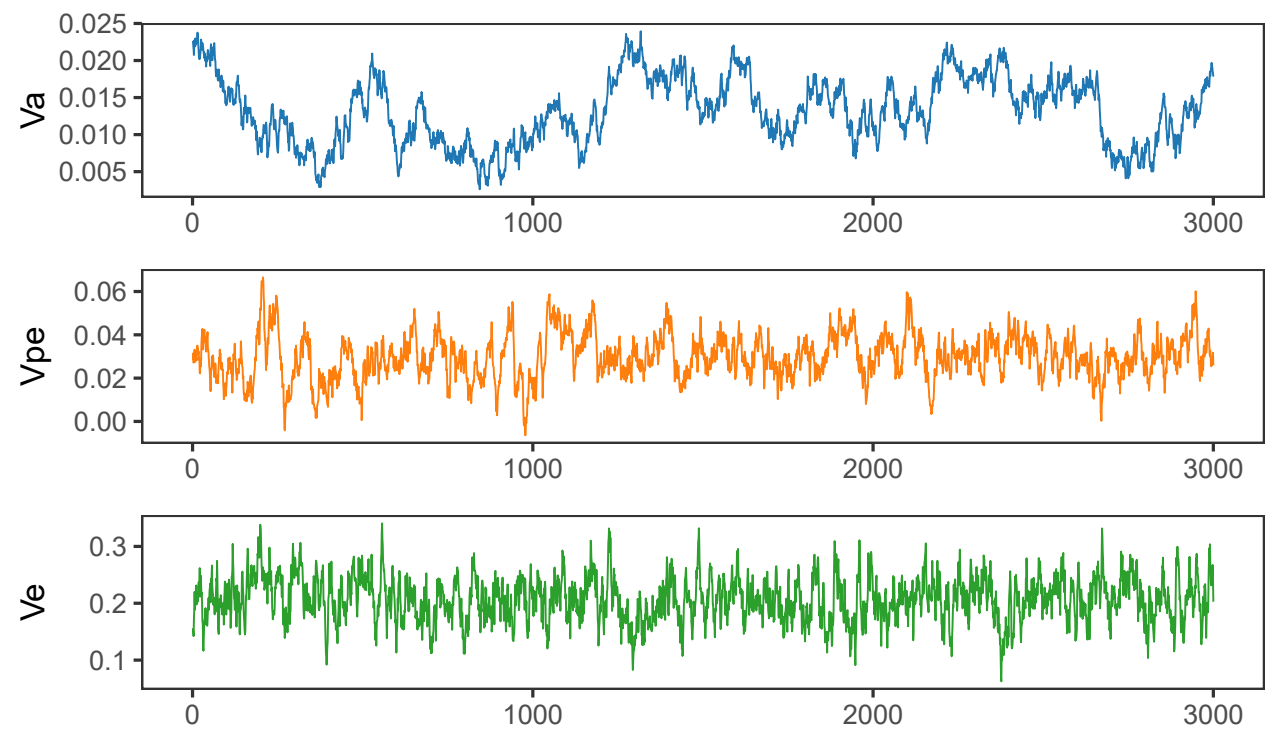


**Figure S2.** Trace plot of variance components for litter size at birth (LSB) from the Poisson model. $V_{a}$, blue: additive genetic variance; $V_{\mathrm{pe}}$, orange: permanent environmental variance; and $V_{e}$, green: residual variance.


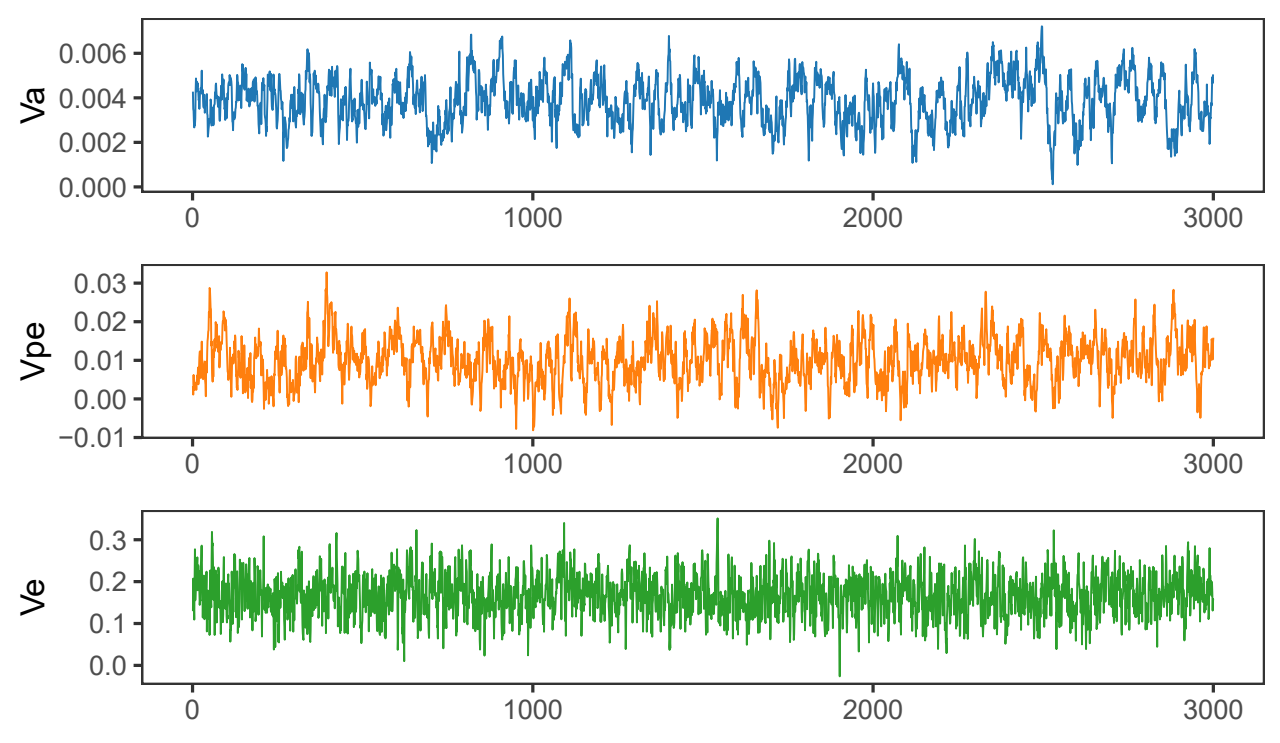


**Figure S3.** Trace plot of variance components for litter size at weaning (LSW) from the linear model. $V_{a}$, blue: additive genetic variance; $V_{\mathrm{pe}}$, orange: permanent environmental variance; and $V_{e}$, green: residual variance.


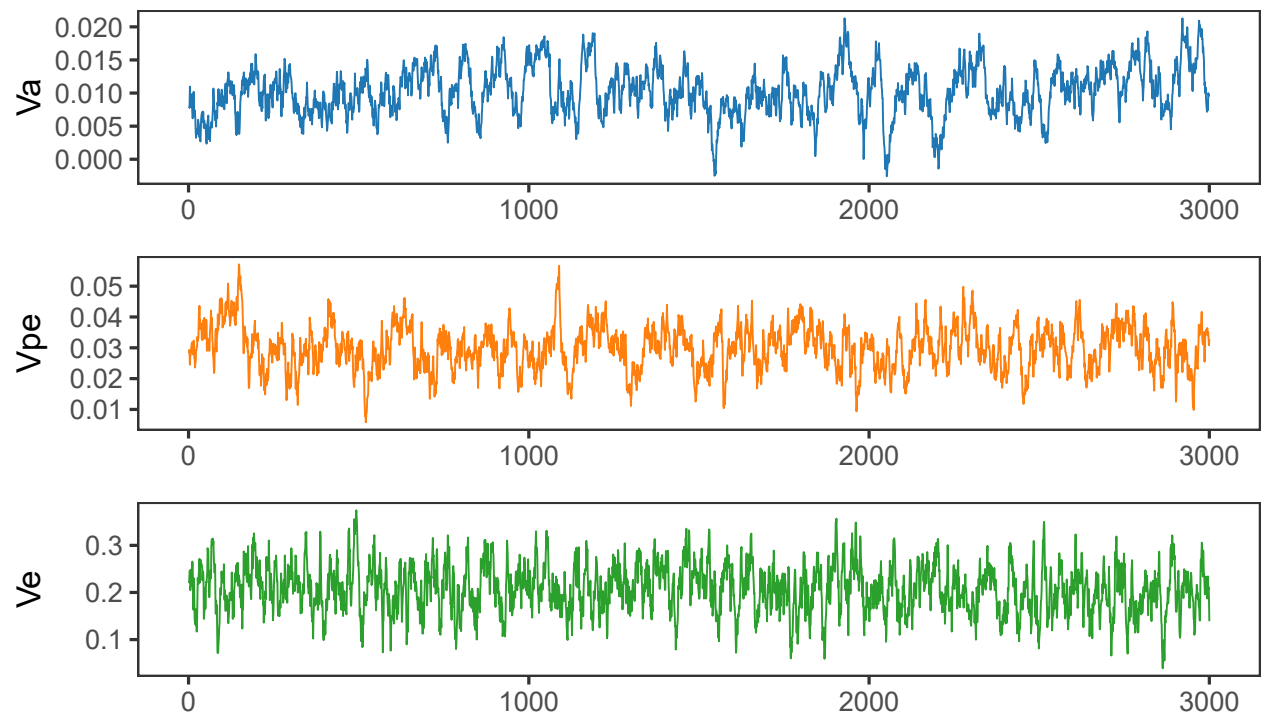


**Figure S4.** Trace plot of variance components for litter size at weaning (LSW) from the Poisson model. $V_{a}$, blue: additive genetic variance; $V_{\mathrm{pe}}$, orange: permanent environmental variance; and $V_{e}$, green: residual variance.


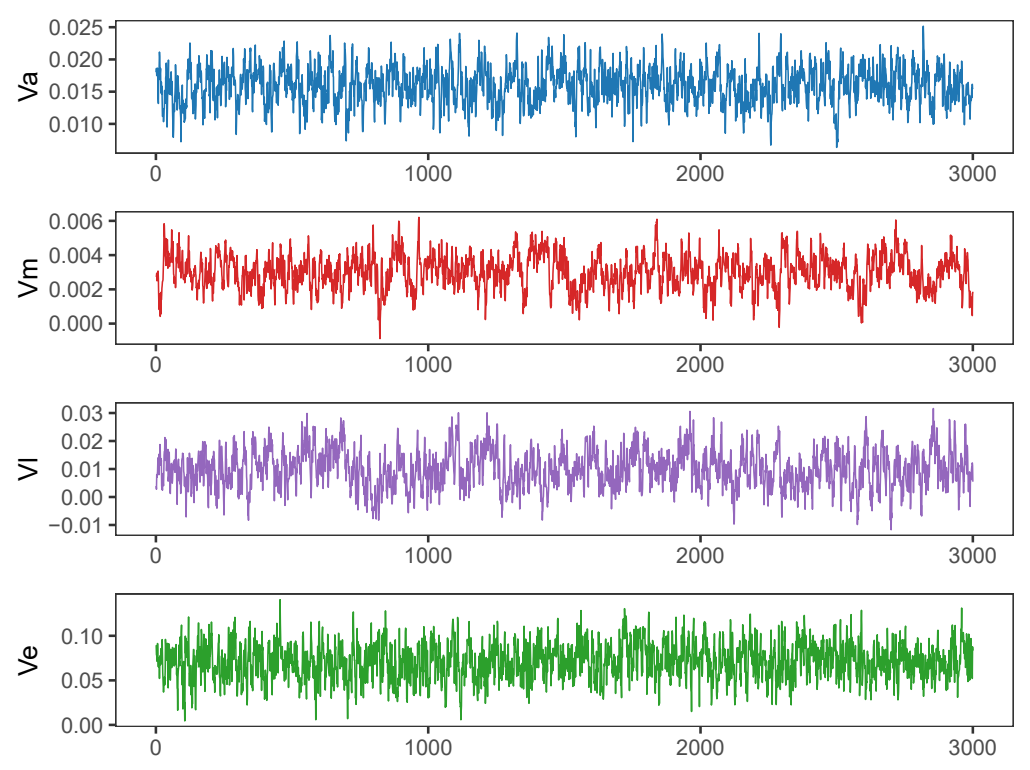


**Figure S5.** Trace plot of variance components for mortality from the linear model. $V_{a}$, blue: additive genetic variance; $V_{m}$, red: maternal genetic variance; $V_{l}$, purple: common environmental (litter) variance; and $V_{e}$, green: residual variance.


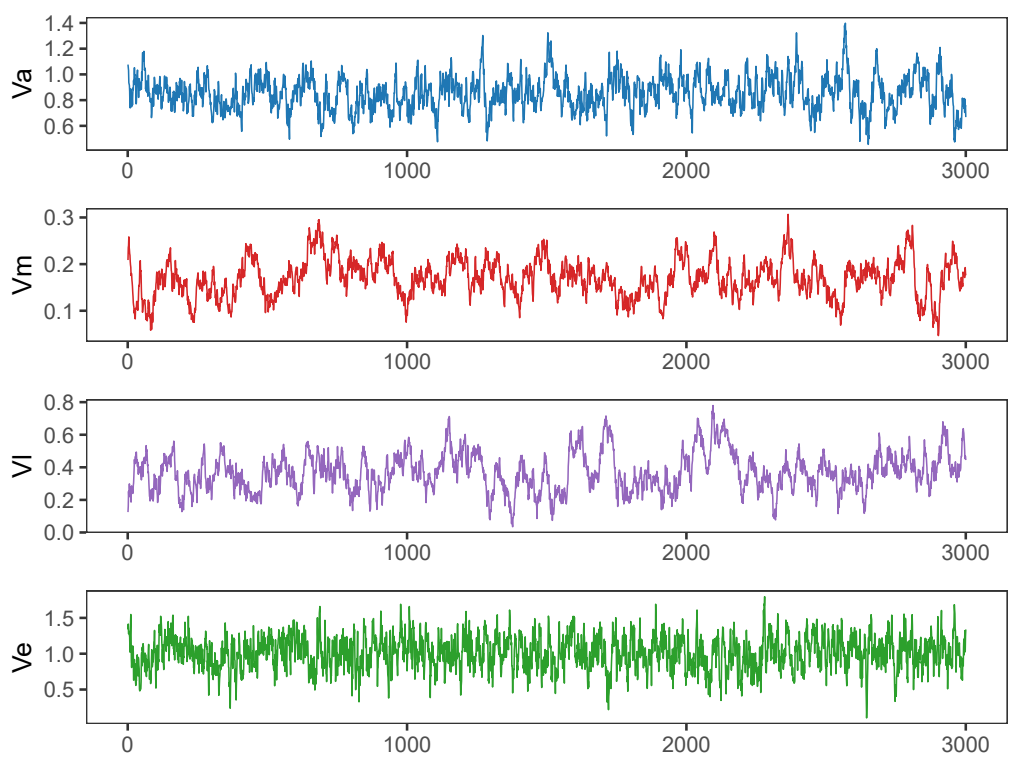


**Figure S6.** Trace plot of variance components for mortality from the probit model. $V_{a}$, blue: additive genetic variance; $V_{m}$, red: maternal genetic variance; $V_{l}$, purple: common environmental (litter) variance; and $V_{e}$, green: residual variance.
